# Cancer Care During the COVID-19 Pandemic: A Retrospective Study From a Najran Oncology Center

**DOI:** 10.7759/cureus.63252

**Published:** 2024-06-26

**Authors:** Ahmed M Badheeb, Sarah H Musallam, Ahlam Y Alyami, Abbas H Almakrami, Ali Dhafer Al-Swedan, Faisal Ahmed, Mohamed Badheeb, Abdullah I Aedh, Hamoud Y Obied, Islam A Seada, Nasher H Alyami, Musadag Elhadi, Abdelaziz A Aman, Samer Alkarak, Hassan K Haridi

**Affiliations:** 1 Oncology, King Khalid Hospital - Oncology Center, Najran, SAU; 2 Oncology, Hadhramout University, Mukalla, YEM; 3 Internal Medicine, King Khalid Hospital, Najran, SAU; 4 Internal Medicine, Najran University, Najran, SAU; 5 Endocrinology, King Khalid Hospital, Najran, SAU; 6 Infectious Disease, King Khalid Hospital, Najran, SAU; 7 Urology, Ibb University, Ibb, YEM; 8 Internal Medicine, Bridgeport Hospital, Yale New Haven Health, Bridgeport, USA; 9 Surgery, Najran University, Najran, SAU; 10 Cardiac Surgery, King Khalid Hospital, Najran, SAU; 11 Cardiothoracic Surgery, King Khalid Hospital, Najran, SAU; 12 Laboratory Medicine, Hematology Unit, Najran General Hospital, Najran, SAU; 13 Medicine, King Khalid Hospital, Najran, SAU; 14 General Surgery, King Khalid Hospital, Najran, SAU; 15 Academic Affairs, King Khalid Hospital, Najran, SAU

**Keywords:** saudi arabia., najran, cancer, health impact, pandemic, cancer care, covid-19

## Abstract

Background

The COVID-19 pandemic significantly impacted healthcare systems globally, with cancer patients representing a particularly vulnerable group. This study aims to evaluate the influence of COVID-19 on cancer, focusing on infection rates, types of care, therapy adjustments, and factors associated with COVID-19 infection.

Materials and methods

This single-center retrospective analysis included adult cancer patients who underwent anticancer therapy at King Khalid Hospital in Najran, Saudi Arabia, from December 20, 2020, to January 23, 2022. Data on patient and cancer characteristics, COVID-19 specifics, treatment delays, outcomes, and factors associated with COVID-19 were collected and analyzed.

Results

A total of 257 chemotherapy recipients were interviewed. The mean age was 52.6 ± 14.4 years, with 44 (17.1%) over 65 years old. Females comprised 160 (62.3%) of the patients. The most common malignancies were gastrointestinal (71, 27.6%), breast (70, 27.2%), and hematological (50, 19.5%). Metastasis was present in 116 patients (45.1%). Common comorbidities included diabetes (68, 26.5%) and hypertension (55, 21.4%). Most patients (226, 87.9%) were vaccinated against COVID-19. COVID-19 tested positive in 22 patients (8.6%), with a lower infection rate in vaccinated patients (7 vs. 15, p < 0.001). Most cases were mild (18, 81.8%), with fever (19, 7.4%) and cough and fatigue (17, 6.6%) being the most common symptoms. The median time to resume treatment post-infection was 30 days. Factors associated with higher infection rates included diabetes (OR: 4.73, 95% CI: 1.94-12.03, p = 0.001), coronary artery disease (OR: 4.13, 95% CI: 1.07-13.30, p = 0.049), chronic lung disease (OR: 15.58, 95% CI: 5.37-45.79, p < 0.001), chronic liver disease (OR: 7.64, 95% CI: 2.38-22.98, p < 0.001), and multiple comorbidities (OR: 2.04, 95% CI: 1.46-2.90, p < 0.001), cancer patients who received chemotherapy (OR: 1.02, 95% CI: 0.12-12.79, p = 0.027), and immunotherapy (OR: 3.37, 95% CI:1.27-8.43, p = 0.012).

Conclusion

The incidence of COVID-19 in cancer patients is proportional to the prevalence in the general population of similar geographic areas. Diabetes, coronary artery disease, chronic lung disease, chronic liver disease, receiving chemotherapy or immunotherapy, and multiple comorbidities were associated with higher COVID-19 infection rates.

## Introduction

COVID-19 has resulted in a pandemic that has affected more than 600 million individuals, with over 6.5 million deaths [1]. Despite these staggering numbers, the incidence of COVID-19 appears to be underestimated due to its diverse clinical presentations, ranging from asymptomatic cases with incidental identification to fatal respiratory and multi-organ failure [2]. Prior reports have highlighted the significantly heightened risk of COVID-19-related complications and mortality among cancer patients compared to the general population [3,4]. Similarly, an increased risk of hospitalization and intensive care unit admissions has been observed in this population [5]. These observations can be attributed, in part, to several risk factors, including the health profiles of cancer patients, who are typically older and have a higher prevalence of chronic cardiopulmonary diseases. Additionally, the immunosuppressive state, resulting from either the illness itself or cancer therapy, further increases the risk for these patients [6,7].

Reports from various regions have underscored the impact of COVID-19 on patient mortality, although there is a notable variation in reported rates. For instance, a Latin American model estimated a mortality rate of up to 30% among cancer patients due to COVID-19 [8]. Similar figures have been reported in the United Kingdom (30.6%) and even higher rates in Central Europe (36.8%) [9,10]. Italian and Chinese studies have also demonstrated a higher risk of COVID-19 and adverse events in cancer patients compared to the general population [11].

Reports from the Middle East, particularly the Kingdom of Saudi Arabia, are limited, leading to a lack of understanding of the incidence rate and risk factors among cancer patients in these regions. This study aims to evaluate the influence of COVID-19 on cancer patients, including the type of care and therapy received, as well as the factors associated with COVID-19 infection in this population.

## Materials and methods

Study design

This study included adult cancer patients who were managed at the cancer center at King Khalid Hospital in Najran, Saudi Arabia, from December 20, 2020, to January 23, 2022, irrespective of the cancer stage and treatment type. All adult patients (aged ≥18 years) who were histologically diagnosed with cancer and actively receiving anticancer therapy for at least one cycle in the last 12 months were included. Patients aged <18 years and those not on anticancer treatment were excluded.

Data collection

Patients were interviewed following outpatient appointments at our center. The demographic characteristics of the patients, including age, sex, indication for anticancer therapy, and primary diagnosis, were obtained from electronic medical records. A survey was conducted on comorbidities such as diabetes, hypertension, coronary artery disease, chronic lung disease, chronic liver disease, presence of multiple comorbidities, cancer site, current cancer status, anticancer therapy, immunosuppressant medication use, history of COVID-19 symptoms or diagnosed infection, history of vaccination and the type and dose of vaccination, history of COVID infection following vaccination, infection severity (based on self-reported symptoms and hospital admission), delay in treatment, and alteration of anticancer therapy based on COVID infection. A confirmed case of COVID-19 was defined as a positive result on a real-time RT-PCR assay of nasal and oropharyngeal swab specimens based on symptomology and a positive laboratory test.

Main outcome

The main objective was to investigate the prevalence of COVID-19 infection and its outcomes. The secondary outcome was to investigate the factors associated with COVID-19 infection.

Statistical analysis

All statistical analyses were performed using SPSS Statistics for Windows, Version 18.0 (Released 2009; SPSS Inc., Chicago, USA). Continuous variables were presented as mean, median, and SD, while categorical variables were expressed as absolute numbers or percentages and compared using chi-square (χ²) or Fisher’s exact tests. ORs and their 95% CIs were derived from β coefficients and standard errors. A two-tailed p-value of less than 0.05 was considered statistically significant.

Ethical approval

This study received approval from the Ethics Research Committees of King Khalid Hospital (Code: KACST, KSA: H-I1-N-089), adhering to the ethical standards set forth in the Declaration of Helsinki. Due to the retrospective design of the study, obtaining written informed consent from the participants was not necessary.

## Results

A total of 257 cancer patients who were actively receiving anticancer therapy were interviewed. The mean age was 52.6 ± 14.4 years (range: 21.0-86.0 years), with 44 (17.1%) older than 65 years. Females comprised 160 (62.3%) patients. Gastrointestinal cancer, breast cancer, and hematological malignancies were the most common cancers (71 (27.6%), 70 (27.2%), and 50 (19.5%) cases, respectively). Metastasis was observed in 116 patients (45.1%). Diabetes and hypertension were the most common comorbidities, occurring in 68 (26.5%) and 55 (21.4%) patients, respectively. The main anticancer treatment was chemotherapy in 203 patients (79.0%), followed by immunotherapy in 38 patients (14.8%) (Table 1). The majority of treated patients (87.9%) began anticancer medication before the initiation of the COVID-19 pandemic and continued it throughout the pandemic. Chemotherapy (79.0%) was administered every week (paclitaxel, gemcitabine, nab-paclitaxel, etc.), every two weeks (FOLFOX-4, FOLFIRI, FOLFOXIRI, etc.), or once every three weeks (FAC, etc.); other treatments included immunotherapy (14.8%), chemoradiotherapy (3.1%), radiotherapy (1.6%), and chemoimmunotherapy (0.8%).

**Table 1:** Characteristics of the cancer patients

Variables	N (%)
Age (year), mean ± SD	52.6 ± 14.4 (range: 21.0-86.0)
Age group	
≤65 years	213 (82.9%)
>65 years	44 (17.1%)
Gender	
Male	97 (37.7%)
Female	160 (62.3%)
Cancer site	
Gastrointestinal cancer	71 (27.6%)
Breast cancer	70 (27.2%)
Hematological malignancies	50 (19.5%)
Gynecological cancer	30 (11.7%)
Thoracic cancers	20 (7.8%)
Genitourinary cancer	16 (6.2%)
Cancer stage	
Non-metastatic	141 (54.9%)
Metastatic	116 (45.1%)
Treatment intent	
Curative	230 (89.5%)
Palliative	27 (10.5%)
Comorbidities	
Diabetes	68 (26.5%)
Hypertension	55 (21.4%)
Coronary artery disease	16 (6.2%)
Chronic lung disease	19 (7.4%)
Chronic liver disease	17 (6.6%)
Multiple comorbidities, mean ± SD	0.7 ± 1.0 (range: 0.0-5.0)
Comorbidity number	
No comorbidity	157 (61.1%)
One comorbidity	50 (19.5%)
Multiple comorbidities	50 (19.5%)

Most patients (226, 87.9%) were vaccinated against COVID-19, and the Pfizer-BioNTech COVID-19 Vaccine was the most common vaccination type used (146, 56.8%). The PCR test for COVID-19 was positive in 22 patients (8.6%). The severity of the COVID-19 infection was mild in the majority of cases (n = 18, 81.8%). The main symptom was fever in 19 (7.4%) patients, followed by cough and fatigue in 17 (6.6%) patients. In most cases, the COVID-19 infection occurred before vaccination (18, 7.0%). Most patients received treatment at home with isolation (13, 59.1%). The median time between COVID-19 infection and treatment resumption was 30 days (min: 30; max: 360 days). In most cases, 13 (59.1%) were improved, eight (36.4%) had a partial improvement, and one case (4.5%) was expired (Table 2).

**Table 2:** Impact of COVID-19 and follow-up of the patients * Some patients had multiple symptoms.

Variables	N (%)
COVID-19 vaccination	
No	31 (12.1%)
Yes	226 (87.9%)
Vaccination type	
Pfizer	146 (56.8%)
Mixed	48 (18.7%)
Astra	28 (10.9%)
Moderna	4 (1.6%)
PCR COVID-19 infection	
Negative	235 (91.4%)
Positive	22 (8.6%)
Time of COVID-19 infection	
Before diagnosis with cancer	4 (1.6%)
After diagnosis with cancer	18 (7.0%)
Main symptoms*	
Fever	19 (7.4%)
Fatigue	17 (6.6%)
Cough	17 (6.6%)
Shortness of breath	16 (6.2%)
Infection severity	
Mild	18 (81.8%)
Moderate	4 (18.2%)
Medical care	
Adamite to the hospital ward	8 (36.4%)
ICU admission	1 (4.5%)
Home isolation	13 (59.1%)
Outcome	
Improved	13 (59.1%)
Partial improvement	8 (36.4%)
Death	1 (4.5%)

Factors associated with the COVID-19 infection

The infection rate was significantly lower in the vaccinated cases than in the non-vaccinated cases (7 vs. 15, p < 0.001). Factors such as diabetes (OR: 4.73, 95% CI: 1.94-12.03, p = 0.001), coronary artery disease (OR: 4.13, 95% CI: 1.07-13.30, p = 0.049), chronic lung disease (OR: 15.58, 95% CI: 5.37-45.79, p < 0.001), chronic liver disease (OR: 7.64, 95% CI: 2.38-22.98, p < 0.001), and multiple comorbidities (OR, 2.04, 95% CI:1.46-2.90, p < 0.001) were associated with COVID-19 infections (Table 3). Additionally, there were treatment delays during the COVID-19 pandemic, especially for positive infection cases, and this relationship was statistically significant (OR: 2.34, 95% CI: 1.52-3.86, p < 0.001).

**Table 3:** Characteristics of patients with and without COVID-19 infection in univariate analysis Note: Boldface indicates a statistically significant result (p < 0.05).

Variables	Subgroups	No COVID-19 infection	COVID-19 infection	OR (95% CI)	p-value
Age (year)	Mean ± SD	52.5 ± 14.5	53.5 ± 13.5	1.01 (0.97-1.04)	0.748
Age groups	≤65 years	195 (83.0)	18 (81.8)	Reference group	1
	>65 years	40 (17.0)	4 (18.2)	1.08 (0.30-3.09)	
Gender	Male	86 (36.6)	11 (50.0)	Reference group	0.312
	Female	149 (63.4)	11 (50.0)	0.58 (0.24-1.40)	
Cancer site	Gastrointestinal cancer	64 (27.2)	7 (31.8)	Reference group	0.181
	Breast cancer	65 (27.7)	5 (22.7)	0.70 (0.20-2.32)	
	Hematological malignancies	43.0 (18.3%)	7.0 (31.8%)	1.49 (0.48-4.64)	
	Thoracic cancers	17 (7.2)	3 (13.6)	1.61 (0.32-6.50)	
	Genitourinary cancer	16 (6.8)	0 (0.0)	-	
	Gynecological cancer	30 (12.8)	0 (0.0)	-	
Treatment intent	Curative	210 (89.4)	20 (90.9)	Reference group	1
	Palliative	25 (10.6)	2 (9.1)	0.84 (0.13-3.12)	
Cancer stage	Non-metastatic	129 (54.9)	12 (54.5)	Reference group	1
	Metastatic	106 (45.1)	10 (45.5)	1.01 (0.41-2.44)	
Diabetes	No	180 (76.6)	9 (40.9)	Reference group	0.001
	Yes	55 (23.4)	13 (59.1)	4.73 (1.94-12.03)	
Hypertension	No	185 (78.7)	17 (77.3)	Reference group	1
	Yes	50 (21.3)	5 (22.7)	1.09 (0.34-2.91)	
Coronary artery disease	No	223 (94.9)	18 (81.8)	Reference group	0.049
	Yes	12 (5.1)	4 (18.2)	4.13 (1.07-13.30)	
Chronic lung disease	No	225 (95.7)	13 (59.1)	Reference group	<0.001
	Yes	10 (4.3)	9 (40.9)	15.58 (5.37-45.79)	
Chronic liver disease	No	224 (95.3)	16 (72.7)	Reference group	<0.001
	Yes	11 (4.7)	6 (27.3)	7.64 (2.38-22.98)	
COVID-19 vaccination	No	26 (11.1)	5 (22.7)	Reference group	0.206
	Yes	209 (88.9)	17 (77.3)	0.42 (0.15-1.37)	
Multiple comorbidities	Mean ± SD	0.6 ± 0.9	1.7 ± 1.5	2.04 (1.46-2.90)	<0.001
Treatment delay (days)	Mean (SD)	3.4 ± 26.5	40.9 ± 38.8	2.34 (1.52-3.86)	<0.001
Time of COVID-19 infection	Infection before vaccination	1.0 (0.4%)	15.0 (68.2%)	-	<0.001
	Infection after vaccination	0.0 (0.0%)	7.0 (31.8%)	-	
	No COVID-19 infection	234.0 (99.6%)	0.0 (0.0%)	-	

Additionally, COVID-19 infection was increased among cancer patients who received chemotherapy (OR: 1.02, 95% CI: 0.12-12.79, p = 0.027) and immunotherapy (OR: 3.37, 95% CI:1.27-8.43, p = 0.012) and were statistically significant. However, it was not among cancer patients who received radiotherapy alone (OR: 0.40, 95% CI: 0.02-6.94, p = 0.577) (Table 4).

**Table 4:** COVID-19 infection among cancer patients according to an anticancer regimen * Some patients had multiple anticancer regimens. Note: Boldface indicates a statistically significant result (p < 0.05).

Anticancer regimen*	Subgroups	Total	No COVID-19 infection	COVID-19 infection	OR (95% CI)	p-value
Radiotherapy	No	245 (95.3)	223 (94.9)	22 (100.0)	Reference group	0.577
	Yes	12 (4.7)	12 (5.1)	0 (0.0)	0.40 (0.02-6.94)	
Chemotherapy	No	44 (17.1)	36 (15.3)	8 (36.4)	Reference group	0.027
	Yes	213 (82.9)	199 (84.7)	14 (63.6)	1.02 (0.12-12.79)	
Immunotherapy	No	217 (84.4)	203 (86.4)	14 (63.6)	Reference group	0.012
	Yes	40 (15.6)	32 (13.6)	8 (36.4)	3.37 (1.27-8.43)	

## Discussion

The COVID-19 pandemic placed an unprecedented strain on healthcare systems worldwide, affecting patients, healthcare providers, and facilities [2,12]. Numerous reports have highlighted the pandemic’s impact on cancer care, including screening, follow-up, and the initiation or continuation of treatment [13]. Additionally, observational studies, including some from Saudi Arabia, have documented the increased vulnerability of cancer patients to COVID-19 and its complications [14]. Therefore, our aim was to assess the impact of COVID-19 on cancer patients, focusing on infection rates, treatment types, delays, and factors associated with COVID-19 infections.

Our study revealed that 8.6% of cancer patients were infected with COVID-19, which aligns with previous reports from the US and a pooled analysis of 58 studies, showing incidences of 7.8% and 8%, respectively [15,16]. There is notable heterogeneity in the incidence and prevalence rates of COVID-19, depending on the study site, demographics, and patient characteristics. Similarly, cancer patients had a relatively variable incidence rate compared to the general population. Our study observed a lower incidence rate of COVID-19 among cancer patients compared to the general population. There are no specific data from Saudi Arabia regarding the prevalence of COVID-19 in cancer patients. However, a recent analysis showed that the prevalence of COVID-19 in Saudi Arabia was 11.9% [17]. A large data pool from the US Veterans Affairs Healthcare reported a lower prevalence of COVID-19 compared to the general population [15]. These findings highlight the uncertainty of COVID-19 prevalence among cancer patients. Furthermore, many reports, including ours, are monocentric and conducted retrospectively, which may increase the risk of heterogeneity and limit generalizability.

Notably, males were disproportionately affected compared to females, similar to older patients. However, these differences were not statistically significant. Our findings align with earlier COVID-19 research conducted worldwide, including studies in the United States, Europe, and China, which have shown different impacts on men and women [18-20]. This variation seems to be attributed to factors beyond exposure. For instance, lower angiotensin-converting enzyme 2 expression might render males more susceptible to infection and more severe presentations [21]. Previous reports have shown that the death rate from COVID-19 increases dramatically with age, and older patients are more likely to be infected [3,22]. Our study might be underpowered due to the small sample size of cancer patients infected with COVID-19 and the higher proportion of younger patients in our sample.

Our study findings indicate that certain comorbidities are significantly associated with COVID-19 infections among cancer patients. Specifically, diabetes (OR: 4.73, 95% CI: 1.94-12.03, p = 0.001), coronary artery disease (OR: 4.13, 95% CI: 1.07-13.30, p = 0.049), chronic liver disease (OR: 7.64, 95% CI: 2.38-22.98, p < 0.001), and having multiple comorbidities (OR: 2.04, 95% CI: 1.46-2.90, p < 0.001) were found to be statistically significant factors. However, hypertension was not associated with COVID-19 in our cohort, possibly due to epidemiological variables related to cancer prevalence [23]. These findings align with previous research, which has established that hypertension, diabetes, cardiovascular disease, respiratory illness, and cancer are associated with an elevated risk of death in COVID-19 cancer patients [6,22]. We did not find any significant variation in COVID-19 rates between patients receiving anticancer medication based on their metastatic status. However, patients with metastasis were more likely to be hospitalized and had a higher likelihood of COVID-19 infection. Similar findings were reported by Ayhan et al. [24]. This may be attributed to the fact that metastatic patients are more vulnerable to infections due to compromised organ function and nutritional deficiencies, which can exacerbate their frailty.

The infection rate was significantly lower in vaccinated patients compared to non-vaccinated patients (7 vs. 15, p < 0.001) in our study. Similarly, Thomas et al. assessed the efficacy of COVID-19 vaccination in cancer patients, revealing an 89.7% vaccine efficacy relative to placebo in a phase III ECC subgroup analysis of 1,647 patients [25]. Additionally, a French prospective multicenter cohort study reported that unvaccinated cancer patients have an increased risk of complications from COVID-19 infection, including hospitalization, mechanical ventilation, and mortality [26]. Collectively, these findings endorse the significant role of the COVID-19 vaccination in mitigating the infection risk.

There was a notable and statistically significant increase in the COVID-19 infection rate among patients who received chemotherapy and immunotherapy. However, this increase was not observed in cancer patients who received radiotherapy alone, as reported in prior studies [13,27]. Contrary to these findings, a recent report by Jee et al. did not show worsening outcomes among cancer patients who received chemotherapy [28]. Similarly, data from the UK did not indicate worse outcomes with the use of systemic chemotherapy in cancer patients infected with COVID-19 [29]. Further research is needed to determine the precise interaction between various antitumor medications and COVID-19 infections.

The COVID-19 infection was associated with delays in the initiation and continuation of cancer therapy in our cohort, with a mean resumption time of 30 days. Similar findings were reported by Kumar et al., who observed a three-week treatment delay among cancer patients with COVID-19 infections [2]. Another study found that anticancer therapy was delayed by 45.86 ± 27.66 days (range: 21-87 days), whereas viral clearance took 25.7 ± 22.68 days (range: 7-79 days) [30]. The rationale for this delay is the requirement for patients to provide proof of COVID-19 clearance before being readmitted to anticancer therapy. Zhang et al. studied the outcomes of patients with COVID-19 and found a more than fourfold higher likelihood of severe events in those who received therapy in the 14 days preceding their COVID-19 diagnosis [31]. Although some reports have shown no worsened outcomes with chemotherapy among cancer patients with COVID-19, it remains unclear if treatment delays have a long-term impact on oncological outcomes in these patients [29].

Study limitations

The study has several limitations, primarily due to the low sample size and disproportionate distribution of patient demographics, which limit its generalizability. Additionally, the retrospective nature of the study means the presented data may be subject to poor or incomplete documentation or loss of follow-up when patients are transferred to other healthcare facilities. We strongly recommend large-scale, multicenter studies to further investigate the significant impact of COVID-19 on cancer patients and to identify factors that might be associated with increased infection risk.

## Conclusions

The incidence of COVID-19 in cancer patients is proportional to the prevalence in the general population of similar geographic areas. Diabetes, coronary artery disease, chronic lung disease, chronic liver disease, receiving chemotherapy or immunotherapy, and multiple comorbidities were associated with higher COVID-19 infection rates.
